# Head and neck cancer patient positioning using synthetic CT data in MRI‐only radiation therapy

**DOI:** 10.1002/acm2.13525

**Published:** 2022-01-19

**Authors:** Emilia Palmér, Fredrik Nordström, Anna Karlsson, Karin Petruson, Maria Ljungberg, Maja Sohlin

**Affiliations:** ^1^ Department of Radiation Physics Institute of Clinical Sciences Sahlgrenska Academy University of Gothenburg Gothenburg Sweden; ^2^ Department of Medical Physics and Biomedical Engineering Sahlgrenska University Hospital Gothenburg Sweden; ^3^ Department of Oncology and Radiotherapy Institute of Clinical Sciences Sahlgrenska Academy University of Gothenburg Gothenburg Sweden

**Keywords:** head and neck, MRI‐only, patient positioning, reference image, synthetic CT

## Abstract

**Purpose:**

The accuracy and precision of patient positioning is crucial in radiotherapy; however, there are no publications available using synthetic computed tomography (sCT) that evaluate rotations in head and neck (H&N) patients positioning or the effect of translation and rotation combined. The aim of this work was to evaluate the differences between using sCT with the CT for 2D‐ and 3D‐patient positioning in a magnetic resonance imaging (MRI)‐only workflow.

**Methods:**

This study included 14 H&N cancer patients, with generated sCT data (MRI Planner v2.2) and the CT deformably registered to the MRI. Patient positioning was evaluated by comparing sCT against CT data: 3D cone beam CT (CBCT) was registered to the deformed CT (dCT) and sCT in six degrees of freedom (DoF) with a rigid auto‐registration algorithm and bone threshold, and 2D deformed digital reconstructed radiographs (dDRR) and synthetic DRRs (sDRR) were manually registered to orthogonal projections in five DoF by six blinded observers. The difference in displacement in all DoF were calculated for dCT and sCT, as well as for dDRR and sDRR. The interobserver variation was evaluated by separate application of the paired dDRR and sDRR registration matrices to the original coordinates of the planning target volume (PTV) structures and calculation of the Euclidean distance between the corresponding points. The Dice similarity coefficient (DSC) was calculated between dDRR/sDRR‐registered PTVs.

**Results:**

The mean difference in patient positioning using CBCT was <0.7 mm and <0.3° and using orthogonal projections <0.4 mm and <0.2° in all directions. The maximum Euclidean distance was 5.1 mm, the corresponding mean (1SD) Euclidean distance and mean DSC were 3.5 ± 0.7 mm and 0.93, respectively.

**Conclusions:**

This study shows that the sCT‐based patient positioning gives a comparable result with that based on CT images, allowing sCT to replace CT as reference for patient treatment positioning.

## 1 INTRODUCTION

In traditional external radiotherapy, computed tomography (CT) images are the primary source of information for treatment planning. Magnetic resonance imaging (MRI) can be incorporated as a secondary source of information, used for delineation of target and organs at risk (OAR) but requires a coregistration between MRI and CT. This coregistration process is associated with systematic uncertainties,^1^, ^2^ caused by imperfect registration algorithms, interscan variation in patient setup, and/or anatomical changes. The head and neck (H&N) region consists of numerous anatomical structures and has a complex freedom of motion, which causes a higher risk of imperfect coregistrations compared to other regions, such as brain.

In the past decade, interest has been directed toward MRI‐only radiotherapy workflows, that is, radiotherapy workflows that use MRI as the only imaging modality. An MRI‐only workflow excludes the CT imaging session, and with this also the anatomical uncertainties derived from the MRI to CT coregistration.^3^ A single imaging session further leads to reduced inconvenience for the patients compared to a multimodality workflow.

Since MRI neither provides the electron densities necessary for absorbed dose calculation nor can be directly reconstructed to digital reconstructed radiograph (DRR) with the same contrast as CT data, additional data processing steps resolving these issues must be incorporated in an MRI‐only workflow. These supplementary steps generally include a conversion of the MRI data to synthetic CT data (sCT). The most common conversion techniques are bulk density assignment, atlas‐ or voxel‐based conversion, and the state‐of‐the‐art approach of today; machine learning.^4^, ^5^, ^6^ In an MRI‐only workflow, sCT and synthetic DRR (sDRR) originating from the sCT data replace the original CT and DRR data.

The accuracy of sCT‐based absorbed dose calculations has been previously evaluated for various sCT generation methods and treatment sites,^7^, ^8^, ^9^, ^10^, ^11^ including the prereleased version for sCT generation in the H&N region used in this study.^12^ Although the dosimetric properties of sCT data generated with various methods have been widely evaluated, not many studies have focused on the accuracy and precision of patient positioning using sCT data.^3^

There are some validations of bone‐based patient positioning using sDRR and/or sCT for brain,^13^, ^14^ prostate,^15^, ^16^, ^17^ rectum,^18^ and pelvis.^19^ However, very few investigations of sCT‐based patient positioning have been performed for the H&N region^20^ and to our knowledge, there are no publications available that evaluate rotations for positioning of H&N cancer patients. There are also no publications available for any anatomical region evaluating the effect of translation and rotation combined. The aim of this work was to evaluate the differences between using sCT/sDRR with the original CT/DRR for patient positioning in an MRI‐only H&N radiotherapy workflow.

## 2 MATERIALS AND METHODS

### 2.1 Patient selection

Patient positioning was evaluated for 14 H&N patient data sets, comprising both sCT data and CT data. Ethical approval was obtained from the Swedish Ethical Review Board (Reg nr. 645‐17). The patients received oral and written information prior to study inclusion and signed a written consent if participating. The inclusion criteria were sufficient field of view (FOV) for both the MRI and CT data to enclose the patient body contour of the H&N and shoulders, no major post‐surgical implants or dental restorations causing disruption of the body contour in the MRI, and cone beam CT (CBCT) as well as orthogonal 2D projections (0° and 90°) for treatment positioning verification.

### 2.2 Images

CT data were acquired using an Aquilion LB CT scanner (Toshiba Medical Systems, Tokyo, Japan). All MRIs were acquired with a 1.5 T Siemens Aera wide bore MR‐system (Siemens Healthcare, Erlangen, Germany), equipped with a flat tabletop (CIVCO Radiotherapy, Iowa, USA). A T1‐weighted Dixon Vibe (3D spoiled GRE) acquisition was utilized for sCT generation using the software MRI Planner version 2.2 (Spectronic Medical AB, Helsingborg, Sweden).^21^ The MRI scan parameters were transversal slices, 448 × 381 scan matrix, 448 × 448 reconstruction matrix, reconstructed in‐plane resolution 1.12 × 1.12 mm^2^, 198 slices, 2 mm slice thickness, no slice gap, 795 Hz/pixel readout bandwidth, 8 ms repetition time, 2.39 ms and 4.77 ms echo time, 3 averages, 3D geometry correction, and a total scan time of 9 min 57 s. Patient setup for MRI and CT acquisition, and the CT scan parameters and the commercially available CNN‐based transfer function estimation algorithm used for sCT generation have been previously described.^12^

CBCT data and orthogonal projections were acquired using the kV On‐Board Imager system mounted on the treatment device (Varian Medical Systems, Palo Alto, CA). The CBCT data had a 512 × 512 reconstruction matrix, 0.91 mm^2^ reconstructed in‐plane resolution, 2‐mm slice thickness and 88 slices. The peak kilo voltage output and exposure were 125 kV and 268 mAs, respectively. The orthogonal projections were obtained at 0° and 90°, and both images had a 1024 × 768 matrix, 0.26 mm^2^ in‐plane resolution, and a peak kilo voltage output and exposure of 85 kV and 5 mAs, respectively.

The CT and MRI were acquired consecutively (within hours), and the CBCT data and orthogonal projections used in this study were acquired at the third treatment fraction, approximately 10 days (8–16 days) after the CT and MRI acquisition. All data were acquired in treatment position, with the patient´s head and shoulders immobilized in a custom‐made five‐point thermoplastic mask (Orfit Industries, Wijnegem, Belgium) using a head support (CIVCO Radiotherapy, Iowa, USA). The CBCT data were acquired for the purpose of this study only and not used for treatment positioning verification. For all treatment instances, including the third fraction where CBCT data were acquired, patient positioning was carried out using bone anatomy in the orthogonal projections.

### 2.3 Initial image registration

The pretreatment CT and MRI images are expected to differ from each other due to slightly inconsistent positioning of the patient or anatomical displacement between the two scanning sessions. For this reason, the CT data were deformably registered to the in‐phase MRI data before further analysis. Prior to the registration, the immobilization mask and background noise were handled by applying −1000 Hounsfield Units (HU) and 0 signal level to the voxels outside the body contours in the CT and MRI, respectively.

For registration, a rigid Euler transformation followed by a nonrigid B‐spline transformation with a local rigidity constrain of the bones (MICE Toolkit, v.1.1.0.0, Nonpi Medical, Umeå, Sweden) was utilized. The registrations were optimized with an adaptive stochastic gradient descent procedure with mutual information as similarity metric, as well as rigidity penalty term for the nonrigid deformation. The deformed CT (dCT) was resampled to obtain the same frame of reference and spatial resolution as the sCT. To validate the physical behavior of the deformation, the determinant of the Jacobian (JD) of the deformation field was calculated within the body contour of the dCT data.^22^

To confirm the anatomical correspondence between the dCT and MRI, the difference in Euclidean distance between anatomical bone landmarks was calculated. The landmarks^23^ are listed in Table 1. The reproducibility of the placement of the anatomical landmarks was evaluated by repeatedly placing the first right landmark 10 times.

**TABLE 1:** List of the bone landmarks used to evaluate the computed tomography (CT) to magnetic resonance imaging (MRI) image preregistration, and the results of the preregistration evaluation

The calculated distances between anatomical bone landmarks	Mean (± 1SD) (mm)
1. Horizontal distance between the medial edge of bilateral mandibular condyles.	0.3 ± 1.2
2. Horizontal distance between the tip of bilateral mastoid processes.	−0.5 ± 2.5
3. Vertical distance between the mentum and the midpoint of the anterior surface of the vertebral body.	−1.3 ± 1.6
4. Vertical mid‐distance of the spinal canal of C2.	0.3 ± 1.7
5. Horizontal mid‐distance of the spinal canal of C2.	0.8 ± 1.0
6. Horizontal distance between the angles of the mandible.	−0.5 ± 1.7
7. Vertical mid‐distance of the spinal canal of C4.	−0.1 ± 1.6
8. Horizontal mid‐distance of the spinal canal of C4.	0.9 ± 1.4
9. Vertical distance between the midpoint of the posterior border of the superior surface of body of hyoid bone to the midpoint of anterior vertebral body.	1.2 ± 2.0

### 2.4 Patient positioning

Patient positioning using 3D CBCT data was conducted in Eclipse Image Registration workspace (Varian Medical Systems, Palo Alto, CA) using the rigid auto‐registration algorithm. The auto‐registration was optimized with a downhill simplex procedure and the mutual information as similarity metric. For each patient, the CBCT data were registered to the dCT and sCT in six degrees of freedom. The volume of interest was selected to cover the vertebral column, mandible, and skull base, and with a bone‐threshold of 200 – 1700 HU corresponding to the clinical practice for 2D patient positioning at our department that relies on the bony anatomy. The registrations were visually inspected and considered to meet the requirements for clinical use, that is, the bony anatomy of CBCT and reference image overlapped.

Patient positioning using 2D orthogonal projections was conducted in Eclipse Offline Review workspace (Varian Medical Systems, Palo Alto, CA). For each patient, the deformed DRR (dDRR) and the sDRR were retrospectively and manually registered to the third fraction´s orthogonal projections in the five available degrees of freedom (2D data cannot be rotated around the inferior–superior axis in the Eclipse Offline Review workspace) by six blinded observers (three radiotherapy technologists [RTTs] and three medical physicists), resulting in a total of 168 registrations. The observers were not informed of the type of DRRs that were registered to the orthogonal projections and were asked to comment on any abnormalities in the reference data.

### 2.5 Evaluations

To assess the 2D‐ and 3D‐positioning accuracy, the difference in displacement in all directions (right–left [R‐L], posterior–anterior [P‐A] and inferior–superior [I‐S], and rotations around these axes) were calculated for dCT and sCT, as well as for dDRR and sDRR. Paired equivalence tests of two one‐sided *t*‐test^24^ was conducted (package Statsmodels v0.10.0, Python v3.7) to verify the equivalence of the mean 2D‐ and 3D‐differences in translation, respectively. The confidence interval was 95%, and the equivalence interval was set to (−1 mm, 1 mm).

**FIGURE 1:**
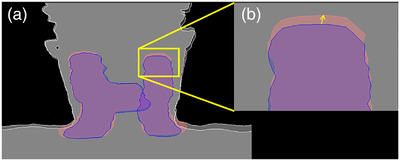
Illustration of the evaluation of interobserver variation. (a) The blue and red areas are the planning target volume (PTV) structure with the deformed digital reconstructed radiographs (dDRR) and synthetic DRR (sDRR) registration matrix applied, respectively. (b) The yellow vector in the cropped image corresponds to the Euclidean distance between a single coordinate in the dDRR‐registered PTV structure and the corresponding coordinate in the sDRR‐registered PTV structure.

The interobserver variation was evaluated by separate application of the paired dDRR and sDRR registration matrices to the original coordinates of the planning target volume (PTV) structures, and subsequent calculation of the difference between the corresponding points (Figure 1). This Euclidean distance was calculated for every point in the DICOM‐RT Struct file (i.e., border of PTV). The Dice similarity coefficient (DSC) was calculated to estimate the overlap between dDRR/sDRR‐registered PTVs for every observer.

## 3 RESULTS

### 3.1 Initial image registration

For all data sets except one, the JDs of the deformation fields were >0 within the body contours. The evaluation of anatomical correspondence of bones gave a maxmimum mean difference of −1.3 ± 1.6 mm for all evaluated anatomical landmarks (Table 1). The 10 repeated distributions of the first right landmark gave a mean and maximum value of 1.5 and 3.4 mm, respectively.

### 3.2 Patient positioning

**FIGURE 2:**
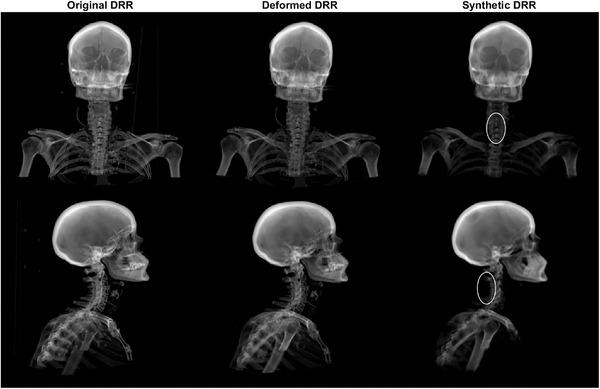
Example of frontal (top) and sagittal (bottom) original digital reconstructed radiograph (DRR) (left), deformed DRR (middle), and synthetic DRR (right). The ellipses show bone structures used for patient positioning that have somewhat decreased contrast and blurred edges in the synthetic DRR.

The sDRRs originating from the sCT data were comparable to the original DRR; however, there were some observable differences in the sDRR such as blurred edges and decreased contrast for part of bone structures (highlighted with ellipses in Figure 2).

### 3.3 2D and 3D positioning accuracy

The mean differences between sCT‐ and CT‐based patient positioning using CBCT were <0.7 mm and < 0.3° in all directions, as seen in Table 2. For all cases, no absolute translation or rotation difference was larger than 2.1 mm and 1.3°, respectively.

**TABLE 2:** The mean (± 1 standard deviation (SD)) difference between synthetic computed tomography (CT) and deformed CT‐based patient positioning, as well as synthetic digital reconstructed radiograph (DRR) and deformed DRR, in all directions

		Translation (mm)		Rotation (°)	
Registration	Direction	mean (± 1SD)	(minimum, maximum)	mean (±1SD)	(minimum, maximum)
3D evaluation using Cone Beam CT	Right‐left/pitch	0.1 ± 0.5	(−1.0, 1.0)	0.3 ± 0.5	(−0.2, 1.3)
	Posterior‐anterior/yaw	0.4 ± 0.7	(−0.7, 1.9)	0.0 ± 0.3	(−0.5, 0.7)
	Inferior‐superior/roll	−0.7 ± 0.6	(−2.1, 0.3)	0.0 ± 0.4	(−0.9, 0.7)
2D evaluation using orthogonal projections	Right‐left/pitch	−0.0 ± 0.7	(−2.5, 1.5)	0.1 ± 0.6	(−1.5, 1.8)
	Posterior‐anterior/yaw	−0.2 ± 0.7	(−2.5, 1.1)	−0.1 ± 0.5	(−1.3, 1.2)
	Inferior‐superior/roll	−0.3 ± 0.8	(−2.5, 1.3)	NaN	(NaN, NaN)

The mean differences between sCT‐ and CT‐based patient positioning using orthogonal projections were <0.4 mm and <0.2° in all directions, as seen in Table 2. For all cases, no absolute translation or rotation difference was larger than 2.5 mm and 1.8°, respectively.

**FIGURE 3:**
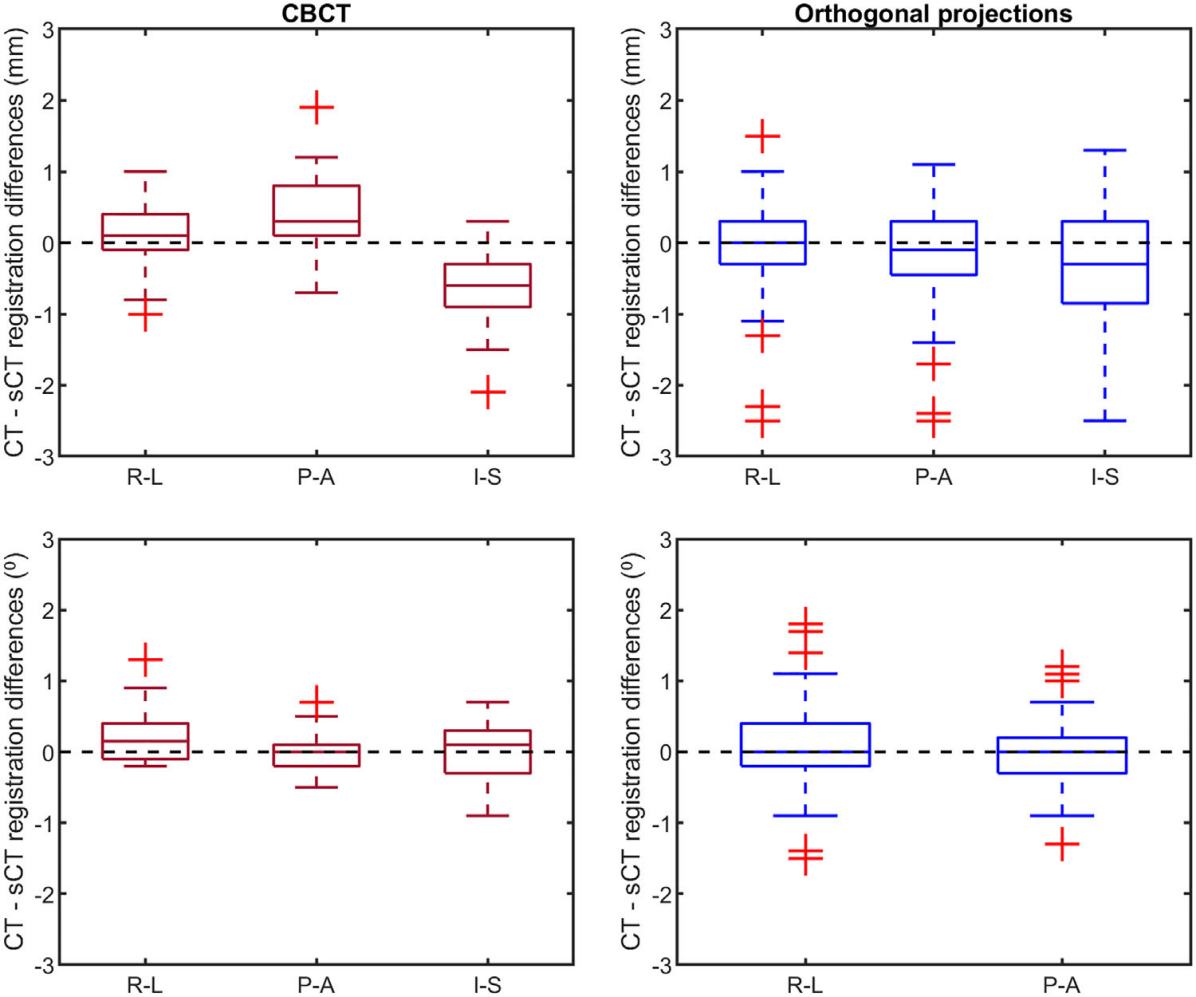
Differences in translation (upper row) and rotation around the specified axis (lower row) between deformed computed tomography (dCT) and synthetic CT (sCT). The red boxplots (left column) show differences in translation and rotation between cone beam CT registered to dCT, and CBCT registered to sCT for 14 cases in the right‐left (R‐L)/pitch, posterior‐anterior (P‐A)/yaw and inferior‐superior (I‐S)/roll directions. The blue boxplots (right column) show differences in translation and rotation between deformed DRR registered to orthogonal and synthetic DRR registered to orthogonal projection for 14 cases and six observers. The bar of the boxplots is the inter‐quartile range (IQR), the whiskers 1.5·IQR and the plus outliers > or < 1.5·IQR.

All translation and rotation differences in each direction between patient positioning using dCT and sCT for both 2D‐ and 3D‐patient positioning are presented in Figure 3.

The paired statistical test of equivalence rejected both t‐tests’ null hypothesizes (*p*‐value <0.001). The translation data fall within the equivalence bonds (−1 mm, 1 mm) for both 2D‐ and 3D‐positioning and are therefore considered equivalent compared to CT.

### 3.4 Interobserver evaluation

**FIGURE 4:**
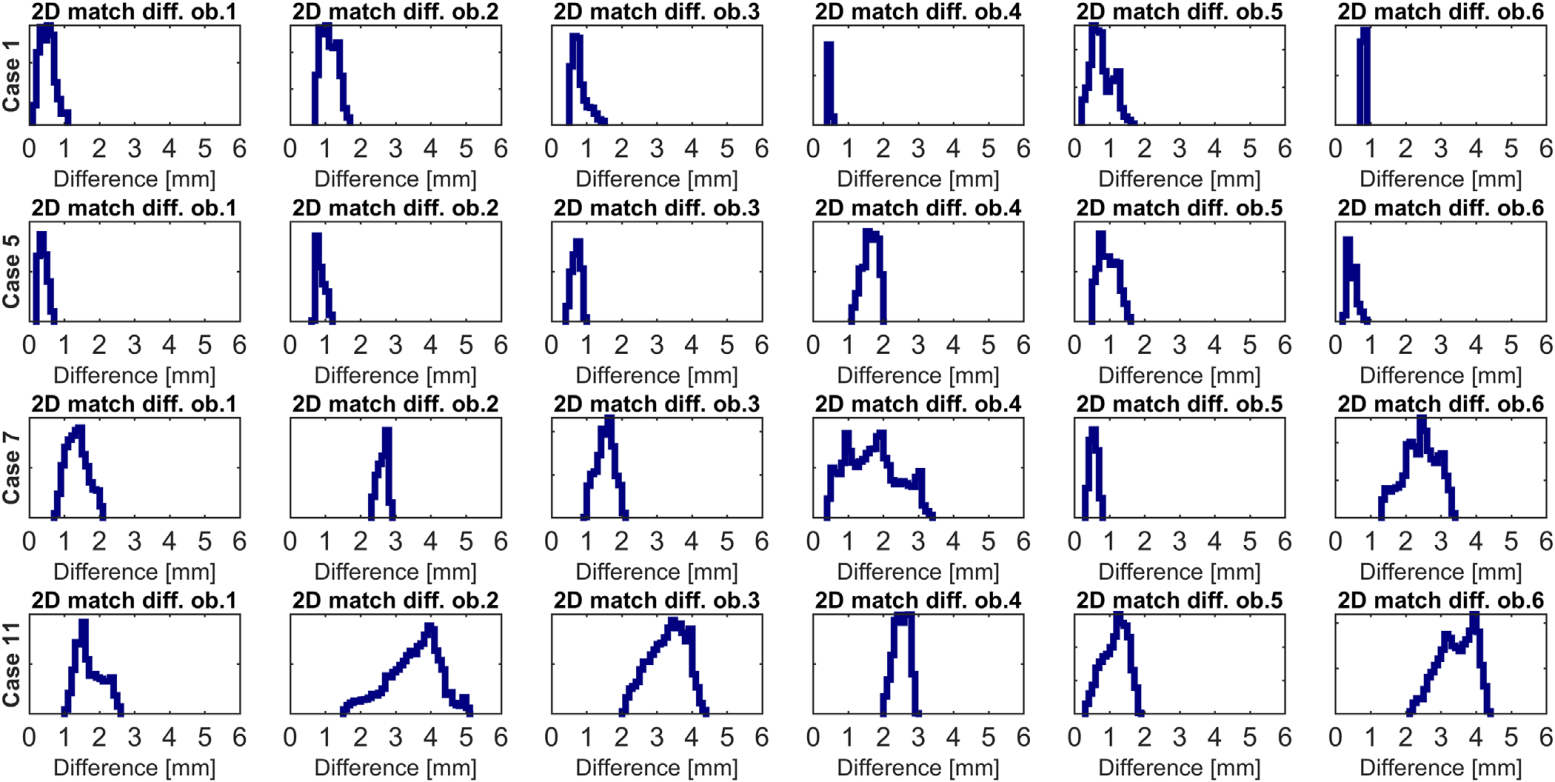
Distributions of the Euclidean distances between every transformed planning target volume (PTV)‐point using deformed digital reconstructed radiographs (DRR) and synthetic DRR‐based registration matrices for the two smallest (case 1 and 5) and largest (case 7 and 11) mean Euclidean distance differences and all observers.

The mean (± 1SD) Euclidean distance for all observers within each case, the maximum and corresponding mean (± 1SD) Euclidean distance for each case of the interobserver evaluation, is presented in Table 3, together with the mean (± 1SD) DSC for all observers within each case. Histograms showing the distribution of distances between every dDRR and corresponding sDRR‐based registered PTV coordinates for the two smallest and largest mean Euclidean distance differences are shown in Figure 4.

**TABLE 3:** The mean (± 1 standard deviation (SD)) Euclidean distance for all observers within each case, the maximum and corresponding mean Euclidean distance of planning target volume (PTV) coordinates found for each case after applying the paired deformed digital reconstructed radiographs (DRR) and synthetic DRR registration matrices to the original coordinates, together with the mean Dice similarity coefficient for all observers within each case

	Euclidian distance			Dice similarity coefficient
	All observers	Worse case observer		All observers
Case	Mean ± 1SD (mm)	Mean ± 1SD (mm)	Maximum (mm)	Mean
1	0.7 ± 0.1	1.1 ± 0.2	1.7	0.99
2	1.2 ± 0.2	1.3 ± 0.6	2.5	0.97
3	1.2 ± 0.2	1.7 ± 0.3	2.8	0.97
4	1.0 ± 0.0	1.4 ± 0.1	1.6	0.97
5	0.8 ± 0.0	1.6 ± 0.2	2.0	0.98
6	0.9 ± 0.2	1.1 ± 0.5	2.1	0.98
7	1.7 ± 0.2	1.7 ± 0.7	3.4	0.96
8	1.4 ± 0.3	1.6 ± 0.6	3.2	0.97
9	1.2 ± 0.1	1.6 ± 0.4	2.4	0.96
10	1.1 ± 0.1	1.8 ± 0.1	1.9	0.98
11	2.6 ± 0.2	3.5 ± 0.7	5.1	0.93
12	1.2 ± 0.2	2.2 ± 0.6	3.5	0.97
13	0.8 ± 0.2	1.2 ± 0.4	2.0	0.98
14	1.5 ± 0.1	2.1 ± 0.7	3.7	0.96

## 4 DISCUSSION

In this study, we have evaluated the use of sCT and sDRR for patient positioning in external radiation therapy by comparing the difference in registration result between sCT/sDRR and the original CT/DRR for the H&N region. The results show that using sCT and sDRR data as reference yields similar registration results as when using CT and DRR data. Even though the sDRR may have some visual dissimilarities in contrast compared to CT‐based DRR, this study shows that they still can be reliably used for patient positioning purposes.

In order to be able to transit from traditional MRI‐CT‐based workflow to an MRI‐only workflow, there are high demands on the image quality of the MRI data used for both the traditional treatment preparation steps (i.e., delineation) and reliable sCT generation. In addition, the combined challenges of the H&N region are complex anatomy, the large MRI FOV required for sCT generation, and the occational presence of surgical implants or dental restorations that cause disruption of the body contour in MRI. MRI can suffer from a large number of artifacts, including metal and motion artefacts, aliasing, chemical shift, etc., which could influence the sCT generation. But apart from motion and dental‐related metal artefacts, no MRI artifacts were observed in this study.

All treatment planning procedures contain systematic treatment preparation errors such as setup errors at the CT and MRI scanner and the treatment unit, delineation errors of the target and OARs as well as the fact that the pretreatment CT and MRI images are instant captures of continuously moving organs.^25^ The strategy used in this study to evaluate differences in patient positioning eliminates some of these errors, such as the initial laser‐based treatment unit setup errors, and delineation error since both images use the same delineated target and OARs. The anatomical differences between the CT and MRI acquisition were mitigated by deformable registration of the CT to the MRI. This is a critical step in this study since a geometrical difference between these images could influence the patient positioning evaluation results.

The physical behavior of the deformation was validated using JD, where a JD greater than 1 indicates expansion of a voxel, and a JD smaller than 1 indicates a contraction. A negative value of the JD would indicate a physically unrealistic deformation for most organs as they only can expand or be compressed, and not undergo noninvertible deformations such as folding.^22^ The JD of the deformation field showed that 13 of 14 dCT data sets had physically realistic deformations (JD > 0). The data set with unrealistic deformation was caused by a physical folding of loose skin at the back of the patient during the MRI acquisition, which was not the case when acquiring the CT images, and hence, cannot be attributed to errors in registration of the MRI and CT images. The landmark evaluation resulted in a mean difference between landmarks in the CT and MRI of approximately 0.3 mm. However, it is essential to recognize that any remaining anatomical difference would systematically affect the results of the patient positioning evaluation. Nevertheless, in an MRI‐only workflow such preprocessing registration will be redundant.

When inspecting the data for case 11, it is clear that the initial registration CT‐MRI is deficient for the vertebra column. The MRI data suffered from motion artifacts over vertebra C3‐C5, causing difficulties in visually distinguishing part of the vertebras. The algorithm generating sCT data appears to be able to generate the body of the vertebras well, but to have some trouble with generating the spinous processes. As a result of the suboptimal deformable registration, differences in 2D and 3D positioning results using sDRR/sCT or dDRR/dCT data were noticeable for this specific case (2D: up to 2.4 mm, 1.7° difference, 3D: up to 2.1 mm, 0.2° difference).

Previous studies have evaluated translational differences for bone‐based patient positioning using synthetic 2D data only,^15^, ^16^, ^20^ and both synthetic 2D and 3D data.^13^, ^19^ There are also studies evaluating both translation and rotation differences using synthetic 3D data.^14^, ^16^, ^18^ To our knowledge, there are no studies evaluating rotations in the H&N region.

Comparing our results with previous studies, the translational differences are similar for patient positioning using orthogonal projections. A maximum mean difference of −0.2 mm in the P‐A direction has been shown for H&N cancer,^20^ compared with our maximum mean difference of −0.3 mm in the I‐S direction. The translational and rotational differences are also similar for patient positioning using CBCT, where a maximum mean difference of 1.5 mm (P‐A) and 1.2° (around R‐L) was shown for prostate.^17^ Both the 2D‐ and 3D‐evaluation had the largest translational differences in displacement in the I‐S directions. This might be the result of a slice thickness of 2.0 mm compared to the reconstructed in‐plane resolution of 1.1 × 1.1 mm.

Until now, adequate setup margins for routine setup verification in H&N radiotherapy have been determined using bone landmarks and 2D kV images^26^ as well as 3D CBCT images.^27^ Previously, it has been concluded that average translational and rotational setup errors of up to 2.2 mm and 0.7° for H&N IMRT treatment plans with a 5‐mm PTV margin, over the entire treatment course, introduce minor dosimetric differences to the CTV (0.39 Gy mean difference of D_98_), PTV (1.21 Gy mean difference of D_95_), and spinal cord (1.20 Gy mean difference of the dose to 1 cm^3^ spinal cord).^28^ Our results were well within these margins and smaller than the setup errors, indicating that utilization of sCT data as reference for patient positioning will have minor impact on the delivered dose distribution.

In our experience, there are no previous studies evaluating both translation and rotation using both 2D‐ and 3D‐data. Evaluating differences in translation and rotation for a reference point is the most common method to evaluate patient positioning. Although, when allowing rotation, one can argue that the impact of a difference in this metric is hard to interpret. We propose employment of Euclidean distances between transformed PTV coordinates using dDRR and sDRR‐based registration matrices in order to interpret this impact. For example, in case 11 – observer 2, the common method resulted in 2.4‐ and 1.9‐mm difference in P‐A and I‐S direction respectively, and 1.7 and 0.9° difference in rotation around R‐L and P‐A. The Euclidean distance method, however, resulted in a mean distance of 3.5 mm and a maximum distance of 5.1 mm between dDRR and sDRR PTV coordinates. The Euclidean distance method also has the benefit of being easily visualized as histograms (Figure 4). Identical rotation between patient positioning using dDRR and sDRR will make the distribution appear as a spike, and an offset of the spike will correspondingly represent a translation offset.

Deviations in patient positioning with orthogonal projections can arise from different observer strategies regarding the use of translation and rotation during image registration, as different registration strategies could provide relatively large differences in translation or rotation between observers and still obtain comparable position of the PTV. The mean DSC found for the different cases shows that the different observers produced registrations that resulted in highly overlapping PTV structures.

Visual analysis of the differences between sDRR and dDRR‐based patient positioning for case 7 pictured different positioning strategies. For the dDRR, all observers aligned the vertebral column in exchange of a slight mismatch for the head. The opposite was found for the sDRR, where the head was aligned, and the vertebral column slightly mismatched. The largest difference between the two modalities was found in the I‐S direction and rotation around R‐L. This was not seen for the CBCT evaluation, where the sCT and CT positioning differences was within the standard deviation for all translation and rotation directions. Visual analyzis of case 14 showed no obvious reason for the deviation, and no trend was seen in any translation or rotation for all observers except one. That single observer had mistaken the GTV guideline for vertebrae guideline, leading to a slightly larger mismatch. Performing a rigid registration in an anatomical region where bone structures are able to move with respect to each other (i.e., flexibility in the neck despite immobilization) could give unclear results since not all structures will be aligned after a successful registration. Therefore, it is difficult to mitigate different registration strategies as an unsuccessful registration. Nevertheless, the mean DSC of 0.96 for both cases shows highly overlapping PTV structures using sDRR or dDRR data.

Using observers for 2D patient positioning evaluation mimics the typical clinical scenario but will also reduce the reproducibility. An alternative to that strategy would be to use an automated patient positioning algorithm. However, these algorithms are not yet widely available, and the positioning still completely relies on the work of RTTs. Other limitations in this study are the low number of landmarks and patient positioning observers, and the number of patients included.

In the future, we intend to investigate the remaining part of the MRI‐only workflow (i.e., delineation) and develop a patient‐specific quality assurance that the generation of sCT brings, with the ultimate goal to treat H&N cancer patient in an MRI‐only workflow.

In conclusion, this study evaluated the use of sCT data as reference for patient positioning using CBCT and orthogonal projections for the H&N anatomy allowing six and five degrees of freedom, respectively. The differences between CT and sCT‐based positioning were comparable, allowing sCT to replace CT as reference for patient positioning at treatment.

## CONFLICT OF INTEREST

The authors have no conflict of interest to declare, except F. Nordström who was previously employed by Spectronic Medical.

## AUTHOR CONTRIBUTION

All the authors listed have contributed directly to the intellectual content of the paper and successfully meets all criteria.

## Supporting information

Supporting Information

## References

[1] Daisne JF , Sibomana M , Bol A , Cosnard G , Lonneux M , Grégoire V . Evaluation of a multimodality image (CT, MRI and PET) coregistration procedure on phantom and head and neck cancer patients: accuracy, reproducibility and consistency. Radiother Oncol. 2003;69(3):237–245. 14644482 10.1016/j.radonc.2003.10.009

[2] Ulin K , Urie MM , Cherlow JM . Results of a multi‐institutional benchmark test for cranial CT/MR image registration. Int J Radiat Oncol Biol Phys. 2010;77(5):1584–1589. 20381270 10.1016/j.ijrobp.2009.10.017 PMC2906611

[3] Jonsson J , Nyholm T , Söderkvist K . The rationale for MR‐only treatment planning for external radiotherapy. Clin Transl Radiat Oncol. 2019;18:60–65. 31341977 10.1016/j.ctro.2019.03.005 PMC6630106

[4] Edmund JM , Nyholm T . A review of substitute CT generation for MRI‐only radiation therapy. Radiat Oncol. 2017;12(1):28. 28126030 10.1186/s13014-016-0747-y PMC5270229

[5] Johnstone E , Wyatt JJ , Henry AM , et al. Systematic review of synthetic computed tomography generation methodologies for use in magnetic resonance imaging‐only radiation therapy. Int J Radiat Oncol Biol Phys. 2018;100(1):199–217. 29254773 10.1016/j.ijrobp.2017.08.043

[6] Owrangi AM , Greer PB , Glide‐Hurst CK . MRI‐only treatment planning: benefits and challenges. Phys Med Biol. 2018;63(5):05TR01. 10.1088/1361-6560/aaaca4 PMC5886006 29393071

[7] Jonsson JH , Karlsson MG , Karlsson M , Nyholm T . Treatment planning using MRI data: an analysis of the dose calculation accuracy for different treatment regions. Radiat Oncol. 2010;5:62. 20591179 10.1186/1748-717X-5-62 PMC2909248

[8] Farjam R , Tyagi N , Veeraraghavan H , et al. Multiatlas approach with local registration goodness weighting for MRI‐based electron density mapping of head and neck anatomy. Med Phys. 2017;44(7):3706–3717. 28444772 10.1002/mp.12303 PMC5510622

[9] Persson E , Gustafsson C , Nordström F , et al. MR‐OPERA: a multicenter/multivendor validation of magnetic resonance imaging‐only prostate treatment planning using synthetic computed tomography images. Int J Radiat Oncol Biol Phys. 2017;99(3):692–700. 28843375 10.1016/j.ijrobp.2017.06.006

[10] Siversson C , Nordström F , Nilsson T , et al. Technical Note: MRI only prostate radiotherapy planning using the statistical decomposition algorithm. Med Phys. 2015;42(10):6090–6097. 26429284 10.1118/1.4931417

[11] Lerner M , Medin J , Jamtheim Gustafsson C , Alkner S , Siversson C , Olsson LE . Clinical validation of a commercially available deep learning software for synthetic CT generation for brain. Radiat Oncol. 2021;16(1):66. 33827619 10.1186/s13014-021-01794-6 PMC8025544

[12] Palmér E , Karlsson A , Nordström F , et al. Synthetic computed tomography data allows for accurate absorbed dose calculations in a magnetic resonance imaging only workflow for head and neck radiotherapy. Phys Imaging Radiat Oncol. 2021;17:36–42. 33898776 10.1016/j.phro.2020.12.007 PMC8058030

[13] Price RG , Kim JP , Zheng W , Chetty IJ , Glide‐Hurst C . Image guided radiation therapy using synthetic computed tomography images in brain cancer. Int J Radiat Oncol Biol Phys. 2016;95(4):1281–1289. 27209500 10.1016/j.ijrobp.2016.03.002 PMC5450663

[14] Edmund JM , Andreasen D , Mahmood F , Van Leemput K . Cone beam computed tomography guided treatment delivery and planning verification for magnetic resonance imaging only radiotherapy of the brain. Acta Oncol. 2015;54(9):1496–1500. 26198652 10.3109/0284186X.2015.1062546

[15] Tyagi N , Fontenla S , Zhang J , et al. Dosimetric and workflow evaluation of first commercial synthetic CT software for clinical use in pelvis. Phys Med Biol. 2017;62(8):2961–2975. 27983520 10.1088/1361-6560/aa5452 PMC5541676

[16] Korhonen J , Kapanen M , Sonke JJ , et al. Feasibility of MRI‐based reference images for image‐guided radiotherapy of the pelvis with either cone‐beam computed tomography or planar localization images. Acta Oncol. 2015;54(6):889–895. 25233439 10.3109/0284186X.2014.958197

[17] Edmund JM , Andreasen D , Van Leemput K . Cone beam computed tomography based image guidance and quality assessment of prostate cancer for magnetic resonance imaging‐only radiotherapy in the pelvis. Phys Imaging Radiat Oncol. 2021;18:55–60. 34258409 10.1016/j.phro.2021.05.001 PMC8254192

[18] Maspero M , Tyyger MD , Tijssen RHN , Seevinck PR , Intven MPW , van den Berg CAT . Feasibility of magnetic resonance imaging‐only rectum radiotherapy with a commercial synthetic computed tomography generation solution. Phys Imaging Radiat Oncol. 2018;7:58–64. 33458406 10.1016/j.phro.2018.09.002 PMC7807733

[19] Kemppainen R , Suilamo S , Ranta I , et al. Assessment of dosimetric and positioning accuracy of a magnetic resonance imaging‐only solution for external beam radiotherapy of pelvic anatomy. Phys Imaging Radiat Oncol. 2019;11:1–8. 33458269 10.1016/j.phro.2019.06.001 PMC7807675

[20] Klages P , Benslimane I , Riyahi S , et al. Patch‐based generative adversarial neural network models for head and neck MR‐only planning. Med Phys. 2020;47(2):626–642. 31733164 10.1002/mp.13927 PMC7146715

[21] Siversson C , Cronholm R , Karlsson A , Brynolfsson J , Adalbjörnss S . White paper: MRI only radiotherapy planning using the transfer function estimation algorithm.

[22] Varadhan R , Karangelis G , Krishnan K , Hui S . A framework for deformable image registration validation in radiotherapy clinical applications. J Appl Clin Med Phys. 2013;14(1):4066. 23318394 10.1120/jacmp.v14i1.4066 PMC3732001

[23] Mohamed AS , Hansen C , Weygand J , et al. Prospective analysis of in vivo landmark point‐based MRI geometric distortion in head and neck cancer patients scanned in immobilized radiation treatment position: results of a prospective quality assurance protocol. Clin Transl Radiat Oncol. 2017;7:13–19. 29594224 10.1016/j.ctro.2017.09.003 PMC5862642

[24] Lakens D . Equivalence tests: a practical primer for t tests, correlations, and meta‐analyses. Soc Psychol Personal Sci. 2017;8(4):355–362. 28736600 10.1177/1948550617697177 PMC5502906

[25] van Herk M , Remeijer P , Rasch C , Lebesque JV . The probability of correct target dosage: dose‐population histograms for deriving treatment margins in radiotherapy. Int J Radiat Oncol Biol Phys. 2000;47(4):1121–1135. 10863086 10.1016/s0360-3016(00)00518-6

[26] Kapanen M , Laaksomaa M , Tulijoki T , et al. Estimation of adequate setup margins and threshold for position errors requiring immediate attention in head and neck cancer radiotherapy based on 2D image guidance. Radiat Oncol. 2013;8(1):212. 24020432 10.1186/1748-717X-8-212 PMC3851546

[27] van Kranen S , van Beek S , Rasch C , van Herk M , Sonke J‐J . Setup uncertainties of anatomical sub‐regions in head‐and‐neck cancer patients after offline CBCT guidance. Int J Radiat Oncol Biol Phys. 2009;73(5):1566–1573. 19306753 10.1016/j.ijrobp.2008.11.035

[28] Fu W , Yang Y , Yue NJ , Heron DE . Dosimetric influences of rotational setup errors on head and neck carcinoma intensity‐modulated radiation therapy treatments. Med Dosim. 2013;38(2):125–132. 23266161 10.1016/j.meddos.2012.09.003

