# Supplementary material for: Head and neck cancer patient positioning using synthetic CT data in MRI‐only radiation therapy
Source: J Appl Clin Med Phys. 2022 Jan 19;23(4):e13525. doi: 10.1002/acm2.13525 (PMC8992936; doi:10.1002/acm2.13525)
Supplement: Supplementary file 1 — Supporting Information [file ACM2-23-e13525-s001.docx]

**Title page**

**Head and neck cancer patient positioning using synthetic CT data in MRI-only radiation therapy**

Emilia Palmér^1^, Fredrik Nordström^1,2^, Anna Karlsson^1,2^, Karin Petruson^3^, Maria Ljungberg^1,2^, Maja Sohlin^1,2^

^1^Department of Radiation Physics, Institute of Clinical Sciences, Sahlgrenska Academy, University of Gothenburg, Gothenburg, Sweden

^2^Department of Medical Physics and Biomedical Engineering, Sahlgrenska University Hospital, Gothenburg, Sweden

^3^Department of Oncology and Radiotherapy, Institute of Clinical Sciences, Sahlgrenska Academy, University of Gothenburg, Gothenburg, Sweden

Corresponding author: Emilia Palmér at Department of Radiation Physics, Institute of Clinical Sciences, Sahlgrenska Academy at University of Gothenburg, MR center, Bruna stråket 13, 413 45 Gothenburg, Sweden, email: [emilia.palmer@gu.se](mailto:emilia.palmer@gu.se)

Running title: Patient positioning using synthetic CT

Author Contribution Statement: All authors listed have contributed directly to the intellectual content of the paper and successfully meets all criteria.

Acknowledgment: The authors would like to thank the clinical staff at the center for all their help and contribution throughout the study.

Conflict of interest: The authors have no conflicts of interest to declare, except F. Nordström who was previously employed by Spectronic Medical.
